# An investigation into the association of pre- and post-migration experiences on the self-rated health status among new resettled adult humanitarian refugees to Australia: a protocol for a mixed methods study

**DOI:** 10.1186/s12914-019-0198-2

**Published:** 2019-04-30

**Authors:** Alison Dowling, Joanne Enticott, Marina Kunin, Grant Russell

**Affiliations:** 1 Department of General Practice, School of Primary Health Care, Monash University, Melbourne, Australia; 2 Southern Synergy, School of Clinical Sciences at Monash Health, Monash University, Melbourne, Australia

**Keywords:** Refugees, Humanitarian, Resettlement, Self-rated health, Longitudinal, Explanatory mixed-methods

## Abstract

**Background:**

Refugees are one of the most vulnerable groups in our society. They are at risk of poor physical and mental health outcomes, much of this attributed to traumatic events prior to migration and the additional risk factors refugees face in the host nations. However, how migration factors shape the health of resettling refugees is not well understood. This study uses a mixed methods approach to examine how pre- and post-migration factors shape the self-rated health of resettling adult refugees in an effort to address the current knowledge gap.

**Methods:**

This study will use a sequential explanatory mixed method study design. We begin by analyzing resettlement and health data from the ‘Building a New Life In Australia’ longitudinal study of humanitarian refugees resettled in Australia to identify significant associations between migration factors and refugee health. Then, a series of semi-structured interviews with resettled refugees will further explore the lived experiences of refugees with respect to the relationship between migration and refugee health. Finally, we will integrate both sets of findings to develop a detailed understanding of how and why migratory factors contribute to refugee health during resettlement.

**Discussion:**

There is a paucity of studies that examine the multidimensional nature of refugee health during resettlement and as a result, little is understood about their resettlement health needs. This information is required to inform existing or new resettlement interventions to help promote or improve refugee health. To overcome these limitations in the research knowledge, this study will use a mixture of study methods to illustrate the complex and multifaceted determinants of refugee health during resettlement in Australia.

## Background

Violent conflicts and persecution are causing increasing numbers of people to be forcibly displaced [1]. Among the 65.6 million people displaced in 2016 are 22.5 million refugees [1]. Refugees who are unable to return to their home country for fear of persecution may be offered resettlement in a third country [1]. Refugees who are accepted for resettlement are among the most vulnerable groups in our society in terms of risk for poor health due to their past and current experiences [2–10]. As one of the current 37 countries worldwide that provide resettlement programs, Australia currently accepts approximately 16,550 refugees each year for permeant resettlement [11].

Many refugees are affected by their pre- and post-migration experiences [12–14]. Most will have witnessed or experienced pre-migration trauma including: torture; human rights violations and systematic violence [15–18]. These events may have a lasting impact on psychological and physical well-being [6, 10]. For example, pre-migration trauma is among the most consistent factors associated with poor mental health in resettled refugees [19, 20]. The time following immigration is often experienced as a time of crisis, stress and adjustment and there is evidence suggesting that resettlement issues, such as housing, employment and financial stress, worsens refugees’ health [19, 21–26]. Therefore, past and current experiences can potentially place refugees at an increased risk for health problems.

Refugee host nations need to understand the health needs of this vulnerable and ever-growing population. However, the impact that migration experiences have upon the health of resettling refugees is not well understood. Some of this knowledge gap can be attributed to methodological limitations in the current research. While qualitative studies of the refugee migration experience provide valuable insights into the meanings of displacement, transition and resettlement [27], it is often difficult to generalise these findings to the broader resettled population [28]. When it comes to the measurement of refugee health, most studies are cross-sectional in design and utilize a variety of quantitative instruments [29]. This has meant that refugee health data is often conflicting and difficult to interpret and compare and can provide only a ‘snapshot’ of a single moment in the refugee resettlement experience. Also lacking are approaches combining longitudinal and qualitative designs that can adequately capture the multidimensional, transitional and contextual elements of the refugee resettlement experience.

Longitudinal studies are needed to detect changes in the characteristics of resettling refugees over time and factors associated with these changes (ie. cause and effect relationship). The refugee resettlement experience is a time of change and resilience, and refugee research must be responsive to such change. Longitudinal studies of resettling refugees can gather important data about the health of refugees, including risk and protective factors and long -term health outcomes. The combination of longitudinal inquiry with qualitative research allows for both measurement and meaning [28]. That is, the qualitative approach gives voice and context to the refugee experience, while the longitudinal approach can be used to provide population-based evidence [28]. This is important because without such information, support services may not be able to effectively deal with the range of challenges that have evolved from the refugee journey and new settlement. Without this knowledge, it is difficult for resettlement nations, such as Australia, to support positive resettlement experiences for refugees and can lead to a significant financial cost to the resettlement nation.

We present a study protocol designed to address these knowledge gaps. The protocol describes a mixed methods approach to identify migration factors that play an important role in the long term refugee health using a combination of quantitative and qualitative data obtained from resettled refugees. Firstly, we will examine pre- and post-migration factors that are associated with self-rated general and mental health using data from a large, longitudinal survey of resettled refugees in Australia. Semi-structured interviews will then be conducted to explain the results of the quantitative study that require further follow up or explanation; such as unexpected findings. To our knowledge, this is the first study of its kind to use the described methodological approach to investigate the health of resettled refugees. It is anticipated that the knowledge that emerges from this study will provide a valuable insight into the health of resettling refugees and lead to recommendations for resettlement services that might be more effective than those currently in place.

## Aim

The overall aim of the study is to identify and explore how pre- and post-migration factors influence self-rated health within adult humanitarian refugees to Australia.

## Research questions

### Quantitative study

Which pre- and post-migration factors are associated with the self-rated health of adult refugees during the first 3 years of resettlement?

### Qualitative study

How do migration experiences influence the health of resettled refugees in Australia?

## Methods

The study will adopt an explanatory mixed methods design using sequential quantitative-qualitative methods [30]. Sequential procedures elaborate or explain the findings of one method with another method [30]. The first phase of the study will involve the secondary analysis of settlement data collected as part of the Australian Government’s ‘Building A New Life in Australia’ longitudinal survey of resettled refugees living in Australia [31]. Statistical analysis of the ‘Building A New Life in Australia’ survey data will be used to identify migration variables that are significantly associated with the health outcomes of resettled refugees. The second phase of the study, namely the qualitative stage, will be designed and carried out for the purpose of interpreting and explaining the results of the quantitative phase using the perspectives of refugees themselves via semi-structured interviews. In the third phase, the quantitative and qualitative data are merged and interpreted. This type of mixed methods study design is used when qualitative data is valuable for explaining or expanding the quantitative findings, particularly in the context where further explanation is required, such as statistical differences among groups or individuals with unexpected findings [32]. In this study, the qualitative portion will explain *how* the migration factors, identified in the quantitative study; play a role in the health of resettling refugees; thereby providing both objective and subjective knowledge of the phenomena under investigation.

**Fig. 1:**
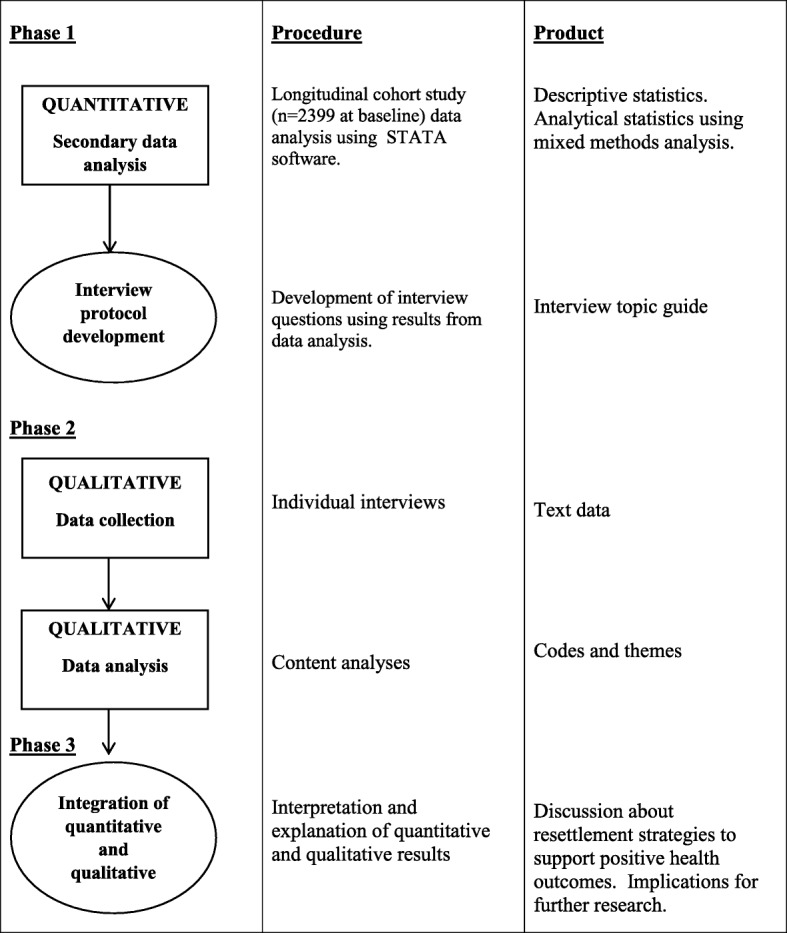
The mixed methods study framework. (Figure adapted from Creswell et al., 2003) [30]

See Fig. 1 for the mixed methods study framework.

### Phase 1: quantitative study

We will examine data from the first three waves of the BNLA study, collected from October 2013 to March 2016. The BNLA is a 5 year national study (2013–2018) conducted by the Australian Government’s Institute of Family Studies to examine how humanitarian refugees settle into a new life in Australia [31, 33]. Details of the ‘Building A New Life in Australia’ study is outlined in existing documents [31, 33] and briefly described below.

#### Ethics approval and consent to participate

The original ‘Building A New Life In Australia’ study was approved by the Australian Institute of Family Studies (AIFS) ethics committee, which is registered with the National Health and Medical Research Council (NMHRC). All necessary administrative permission to use the “Building A New Life in Australia” data have been obtained by the study authors. De-identified BNLA data is accessible by authorized researchers who have obtained permission from the Australian Department of Social Services. This permission was obtained by study authors AD, JE and GR. Ethics exemption to use the data was granted by the Monash University Research Ethics Committee.

#### Consent for publication

Obtaining individual consent from participants to use their de-identified data was not required by the study authors and ethics exemption to use the data was granted by the Monash University Research Ethics Committee.

#### Participants

In this part of the study, no participants will be recruited or contacted by the researchers. Instead, we will conduct a secondary analysis of the sample recruited for the ‘Building A New Life in Australia’ study. To be eligible for the ‘Building A New Life in Australia’ study, individuals needed to be aged 15 years and over and had been granted a permanent humanitarian visa by the Australian Government in the 3–6 months preceding the baseline survey [31]. The respondents are from 35 different countries and speak 50 different languages. At baseline, the ages of respondents ranged from 15 to 83 years [31].

#### Sampling

Participants for the ‘Building A New Life in Australia’ study were drawn from the Australian Department of Immigration and Border Protection settlement database from 11 locations around Australia. Migrating Units (MU) were the primary sampling units for the ‘Building A New Life In Australia’ study [31, 33]. Migrating Units consisted of principal applicants (PA) and secondary applicants (SA). Principal Applicants were the lead participant for the study, and were the initial individuals contacted for participation. Secondary Applicants are other members of the Migrating Unit noted on the Principal Applicant’s visa application (eg. spouse or child) [31, 33].

#### Recruitment

Invitations to participate in Wave 1 of the study, in English and primary Principal Applicant language, were sent to the Principal Applicants. Bilingual Community Engagement Officers, also employed to assist researchers recruit for Wave 1; liaised with community leaders to locate individuals and provide a bilingual perspective to optimise recruitment of study participants. After Principal Applicant agreement to participate, Secondary Applicants within consenting Migrating Units invited to participate. Invitations to participate in waves 2 and 3 were extended to participants in the previous wave, via mail and telephone [31].

#### Measurement

This study will utilise data collected via the ‘Building A New Life in Australia’ survey and covers a range of key domains relevant to refugee resettlement (eg. demographic, housing, pre-migration experiences, health) [31]. The ‘Building A New Life in Australia’ survey was translated into 14 different languages with the availability of interpreters [31].

#### Data collection

The first three waves of ‘Building A New Life in Australia’ data were obtained using alternating waves of home visits (Waves 1 and 3) and telephone interviews (Wave 2) [31].

#### Variable selection

**Fig. 2:**
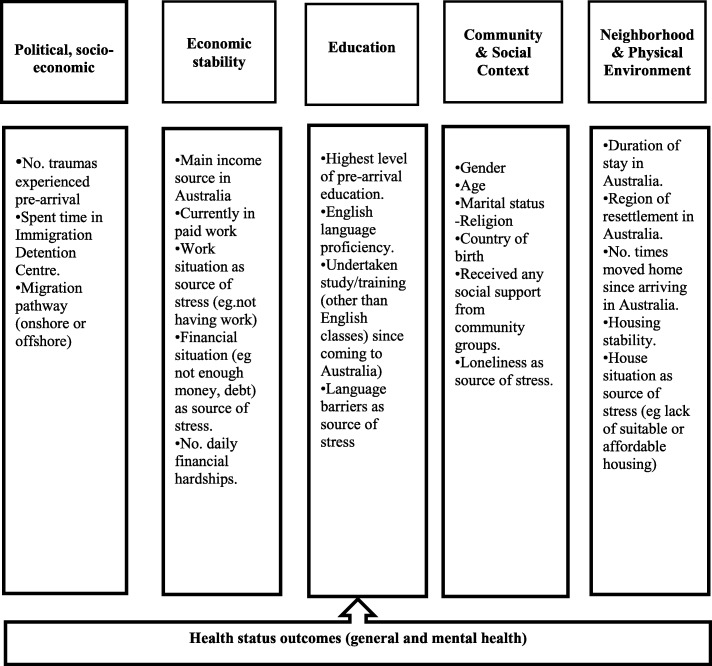
Conceptual framework for the social determinants of health in resettled refugee populations

Variable selection for this study was informed by a conceptual framework oriented to Cross-Denny & Robinson’s social determinant of health model (SDOH) [34]. A review of the literature reporting significant predictors of refugee health outcomes informed the selection of candidate variables from the ‘Building A New Life in Australia’ dataset to populate the social determinant of health model. The model includes five key areas or determinants of refugee health: Political and Socio-economic, Economic stability, Education, Community and Social Context and Neighborhood and Physical Environment. See Fig. 2 for the social determinant of health model used to frame this study. Only variables asked across all three waves will be included in this study.

#### Outcome variables

We have four outcome variables: 1) general health; 2) severe mental illness; 3) Post Traumatic Stress Disorder (PTSD); and 4) aggregated mental health, combining 2) and 3).

1. The general health status item used in the BNLA study is “Overall, how would you rate your health during the past 4 weeks?” and is taken from the 36-Item Short Form Health Survey (SF-36) [35]. The SF-36 is a generic measure of self-reported mental and physical health status in past month. Shorter versions of the SF-36, in which this single item are contained, have reliability and validity among refugee populations [29]. Responses in the BNLA survey are scored on a 6-point Likert scale ranging from “Excellent” to “Very Poor”. A dichotomous variable will be created and comprise of two response categories of “Excellent-good” health and “Fair-Poor” health.
2. Severe mental illness experienced in the past 4 weeks is measured in the ‘Building A New Life in Australia’ survey using the Kessler Screening Scale for Psychological Distress (K6). The Kessler-6 (K6) is a simple six-item measure of non-specific psychological distress [36]. In the ‘Building A New Life in Australia’ survey, the K6 scores range from 9 to 30 and a score ≥ 19 is used as a cut-off for the positive outcome of ‘severe mental illness’ [31]. While the validity of the K6 is not yet formally established in refugee populations, it has good reliability among refugee groups [29] and is well-validated among non-refugee populations [37]. Our study will use a dichotomous variable indicating the presence or absence of probable serious mental illness.
3. Post Traumatic Stress Disorder (PTSD) is measured in the ‘Building A New Life in Australia’ survey using the PTSD-8 [38]. The PTSD-8 is derived from Part 4 of the Harvard Trauma Questionnaire [39], which is a longer measure of trauma symptoms specifically designed for use in refugee populations. Respondents are asked how much each symptom has bothered them a) since the trauma and, if yes, b) in the past month. Items are answered on a 4-point scale (“not at all” to “extremely”). Respondents who answered “sometimes” or “most of the time” for at least one item in each PTSD domain are classified as having PTSD [31]. The PTSD − 8 has both reliability and validity among refugee populations [29].
4. An aggregate ordinal measure combining both serious mental health and PTSD outcome variables will also be examined. This variable will be coded to comprise of three response categories indicating the presence or absence of ‘mental illness’ and/or ‘PTSD’ across a given wave. (That is, “neither serious mental illness or PTSD present”; “serious mental illness present, but not PTSD”; “PTSD present, but not serious mental illness”; “both mental illness and PTSD present”.

#### Data analysis

Statistical analysis will include descriptive and modelling stages. Descriptive statistics, such as frequencies and percentages, will be used to describe the overall characteristics of the sample population, such as socio-demographics, migration experiences and self-rated health outcomes. Modelling will be used to guide variable selection, examine relationships longitudinally between categorical outcomes and predictor variables. A variable exclusion process will be applied in several steps. Firstly, univariate ordinal regressions will be used to examine associations between baseline predictor variables and outcome variables, with variables retained at *p* < 0.1. Secondly, collinearity will be examined using Pearson’s correlation coefficient and one candidate variable will be selected from two or more collinear variables when r > 0.5. This will ensure that variables giving the same information will be excluded from the final model. Third, multivariate panel data models will be used to examine associations between candidate variables and the primary outcome measures longitudinally. Variables at *p* < 0.1 will be retained. Finally, candidate variables will be modelled over 100 bootstrap samples at 95% resampling for the data set. Candidate variables will be selected for use in final models if they are identified as significant in 100 bootstraps.

Mixed effects models will be used to examine associations between predictor variables and outcome variables. As the Migrating Unit was the primary sampling unit for the ‘Building A New Life in Australia’ study, the sampling method was clustered and therefore appropriate for analysis with mixed effects modelling. The mixed effects model account for *inter-*individual random effects *between* different families (Migrating Units) and individual participants [40]. Mixed effects modelling also account for *intra-*individual fixed effects; repeated measures *within* individuals [40].

Stata/SE 15.1 will be used for all analysis [41]. All analyses will use adjusted weights unless otherwise specified.

### Phase II: qualitative study

The second phase of this study, the qualitative research, will be used to interpret or reflect on the quantitative findings in terms of deeper understanding of what meanings and reasoning refugees attribute to their health during resettlement, in particular with regard to their migration experiences.

#### Ethics approval

Permission to carry out the qualitative study and its design has been granted by Monash University’s Human Research Ethics Committee: Approval Certificate Number 12616. All participants in the qualitative study will be required to provide written informed consent prior to the interviews.

#### Conceptual framework

A qualitative phenomenological approach will be used in this research to understand the experience of health of resettled refugees in Australia. It will explore the meaning of health for resettling refugees as reflected in their thoughts on health and migration. Understanding their perspectives will help to elucidate further the explanatory models of this population group and develop evidence based strategies to improve health services for this population.

#### Recruitment

A purposive criterion sampling strategy will be used to recruit participants for this study as this approach will allow us to select respondents based on the shared experience of becoming a refugee who has migrated to, and resettled in, Australia. The sample size for this study will follow the concept of saturation [42]. That is, we will recruit participants until the collection of new data does not provide any further information that will assist in our answering the research question. We anticipate that a minimum of 10 interviews will be required before saturation starts to occur.

Participants will be recruited through centers that provide adult migrant English language programs to refugees located in Melbourne. Permission will be obtained from each center to allow the students to be approached. The primary researcher will visit classes to speak to the students about the study and students will be invited to participate in the study. The primary researcher will then work with potential participants/participants to organize another time to meet with them for interview at a location (eg. a private room at the English learning center or their home) and time suitable to them, (eg. prior to or after their English class).

#### Inclusion criteria

Refugees who participate in English language classes in Melbourne will be eligible to participate in the study. The rationale behind our recruitment strategy is that humanitarian refugees who resettle in Australia are offered 510 h of free English language tuition [11]. In addition, those on humanitarian visas must meet the following time frames from the date of visa commencement or arrival in Australia to remain eligible: 1) they must register within 6 months of receiving their protection visa; 2) start studying within 12 months and 3) finish studying within 5 years [11]. Therefore, humanitarian refugees living in Australia are required to undertake and complete English language classes. Other inclusion criteria for the study will include individuals who:

1. aged over the age of 18 years;
2. have been permanently resettled in Australia for ≤ 4 years;
3. have sufficient English language competency as assessed by general ability to hold a conversation and read basic English; and
4. are able to provide informed consent to participating in the study

#### Data collection

Semi-structured, one-on-one interviews will be used to examine the migration and health experiences of participants. The use of semi-structured interviews is suitable for this study as they are designed to draw out the refugee narratives of the significant domains of the refugees’ experience of health and migration. The explication of these narratives will allow us to gain a better understanding of the ways in which these experiences are perceived, encountered and negotiated within refugee populations [43]. Such knowledge can be used to ensure that interventions designed for resettled refugee communities are appropriate and are targeting those variables considered most critical by the refugees themselves [43].

Participants will be invited to be interviewed individually to capture the experience of health and migration. Semi-structured interviews will be conducted at a venue of the participant’s choosing (e.g. home, English learning centre). The interview data will be recorded through tape recording and note taking.

#### Measures

The data will be collected using an interview designed using the results of the quantitative study. The interview will further probe significant or anomalous results obtained from the quantitative study regarding the relationship between migration factors and health status.

#### Data analysis

Data analysis in this study will be informed by the recommendations for phenomenological and qualitative analysis set forth by Creswell [30]. All interviews will be transcribed verbatim and then checked for accuracy. The data analysis will involve the identification of themes that are considered the respondents’ expressions of the salient experiences of their health and migration, as well as their concerns [30]. This process involves two steps. The first step involves open coding where interview transcripts are read several times and key issues mentioned by respondents are noted [30]. The second step will involve selective coding where phrases, statements, and comments are labelled and categorized according to their content. The second stage involves identifying connections between the codes identified in stage one so that emergent themes can be identified [30]. The identification of emergent themes occurs by noting similarities and differences in the content of the respondents’ statements [30]. This is then followed by translating the emergent themes into a narrative account of the experiences of the participants [30].

### Phase III: mixed methods study

Mixed methods research provides strength to offset the weaknesses of both quantitative and qualitative research [44] and is well suited the current study as it can ensure a rigorous examination of the association of migration factors and refugee health. A mixed methods research design will enable us to utilize multiple methods of data collection and analysis. This will enable a comprehensive study of our complex study of migration and refugee health [44]. Further, a mixed method framework will help us answer questions that cannot be answered by a singular quantitative or qualitative study [32]. While the quantitative phase will allow us to identify *what* migration factors are associated with health outcomes among resettling refugees, it will not explain or elaborate why (or how) this association occurs and relies on qualitative explanations from the community under investigation. Therefore, both methodologies will complement and extend each other, by addressing the research questions from different perspectives. In this way, the study design is sequential and explanatory (or connected) [30].

In this study, the authors will connect the quantitative and qualitative data by selecting the participants for the qualitative interviews using similar criteria to the quantitative study. This will ensure that comparisons are able to be made across the two study phases in terms of the information collected from the study participants. Furthermore, as the findings from the quantitative study will be used to develop a framework for the topic guide for the subsequent interviews, integration will flow from quantitative data analysis into qualitative data collection. For example, any significant, non-significant or surprising quantitative results that require explanation will be used in the development of the qualitative interview guide.

In this way, the themes from the qualitative study will explain the quantitative results in a manner that could not be accomplished using either study method alone.

## Discussion

This explanatory mixed methods study design in refugee health research is an approach to examine the association of migration experiences on the self-rated health status of resettled humanitarian refugees in Australia. It is more comprehensive than previous studies due to a quantitative and longitudinal analysis of refugee health determinants using a large and ethnically diverse cohort of resettled refugees living in Australia. This inquiry will reveal significant health factors and health outcomes among refugees within the initial stages of resettlement. For example, post-migration unemployment rates among resettled refugees are typically high [45] and studies have shown a strong positive association between unemployment and mental illness among refugee populations. However, the mechanism of employment in relation to mental illness among refugees is unknown. Evidence as to whether it alleviates economic stress, provides a sense of purpose or is a source of community support is lacking in the literature. Such insights could be gained from explanations derived from the refugees themselves. Further, this information would provide a useful framework for the provision or improvement of refugee resettlement programs, such as job skills and literacy programs.

It is only once we understand the determinants of refugee health that effective interventions can be provided. The combination of quantitative and qualitative methods will give new and rich insights into the health of resettling refugees as navigate their way into a new life. Such information is important so that we understand the range of adversities refugees face and their relation with mental and physical health symptoms, as well as how health changes from the point of arrival and across years of residence in the host country. This knowledge could build an evidence base for development of improved settlement policies and programs, such as targeted health care and health promotion. It is also intended that this protocol will contribute to the awareness among researchers of the value of mixed methodologies in future refugee health research.

## Ethical considerations

Research involving refugees as study participants poses unique ethical challenges. Refugees can be vulnerable, particularly when engaging as subjects in research [46]. Ethical concerns can include how refugees may be harmed or benefited by research; what they might understand or not about the research process, and to what extent they will feel able to make a free choice [28]. Some of the key methodological challenges of research with refugee populations are developing appropriate research approaches with populations with a range of literacy skills and languages, who are unfamiliar with research and have diverse migration experiences. In addition, research must not add to the burdens of resettlement, and should ideally contribute to a positive experience of adjustment to a new country. To minimize such challenges, the current study has been designed with a number of ethical considerations in mind. These are discussed below.

The recruitment strategy for the qualitative study will focus on settings, such as English migrant learning centers, because it is anticipated that potential participants may already feel some sense of belonging, security and trust with the these center(s). Potential participants will be chosen by the center staff to ensure that only students with sufficient English language competency are approached. To address any difficulties with disclosure and comprehension, all participants will receive a short oral presentation about the study. This talk will be given by the first author at each recruitment site so that a relationship with potential participants can be established. Also, any questions about the study can be addressed at the time. To ensure all potential participants have the capacity to make an informed decision about participating in the study, they will be given detailed written information on the study, which informs them that they will be asked to sign a consent form indicating their willingness to participate and attend an interview. Translated copies of study documentation will be also available to participants. Where required, translators will be available to attend the interviews. All interpreters will be briefed around issues of confidentiality.

To minimize the likelihood of distress among participants, the interviews have been to ensure that participants are not asked to disclose any details of any past traumatic traumas experienced. The questions are designed so that participants provide as much or as little information as they feel comfortable providing, regarding their migration experiences. The participant Information Sheet provides information regarding appropriate counselling and support services that can be accessed by participants should they feel distress or discomfort at any time during their participation in the study or interview. Participants will also be informed that they can stop the interview until such time that they wish to continue. Participants will be given a $20.00 gift card to acknowledge the time and effort involved in participating in the study and to offset any travel costs.

## Abbreviations

BNLA: Building A New Life In Australia
K6: Kessler Screening Scale for Psychological Distress
MU: Migrating Units
NMHRC: National Health and Medical Research Council
PA: Principal applicants
PTSD: Post Traumatic Stress Disorder (PTSD)
PTSD-8: Posttraumatic Stress Disorder Inventory- 8 items
SA: Secondary applicants
SDOH: Social determinant of health model
*SF-36*: *36*-Item Short Form Health Survey

## References

[1] United Nations High Commissioner for Refugees: Global Trends, 2016. Geneva. http://www.unhcr.org/globaltrends2016/ Accessed 25 Jan 2017.

[2] Fazel M, Wheeler J (2005). Prevalence of serious mental disorder in 7000 refugees resettled in western countries: a systematic review. Lancet..

[3] Mollica R, Sarajlic N (1987). Longitudinal study of psychiatric symptoms, disability, mortality, and emigration among Bosnian refugees. JAMA..

[4] Silove D, Steel Z (1998). Trauma exposure, postmigration stressors, and symptoms of anxiety, depression and post-traumatic stress in Tamil asylum-seekers: comparison with refugees and immigrants. Acta Psychiatr Scand.

[5] Cunningham M, Cunningham JD (1997). Patterns of symptomatology and patterns of torture and trauma experiences in resettled refugees. ANZJP..

[6] Steel Z, Silove D (2009). International and indigenous diagnoses of mental disorder among Vietnamese living in Vietnam and Australia. Br J Psychiatry.

[7] Benson J, Phillips CB (2015). Low levels of vitamin B12 can persist in the early resettlement of refugees: symptoms, screening and monitoring. Aust Fam Phys.

[8] Johnston V, Smith L (2011). The health of newly arrived refugees to the top end of Australia: results of a clinical audit at the Darwin refugee health service. Aust J Prim Health.

[9] Gerritsen A, Bramsen I (2006). Physical and mental health of afghan, Iranian and Somali asylum seekers and refugees living in the Netherlands. Soc Psych Psych Epid..

[10] Porter M, Haslam N (2005). Predisplacement and postdisplacement factors associated with mental health of refugees and internally displaced persons: a meta-analysis. JAMA..

[11] Australian Government Department of Home Affairs: Fact sheet - Australia's refugee and humanitarian programme. https://www.homeaffairs.gov.au/about/corporate/information/fact-sheets/60refugee. Accessed 13 Mar 2017.

[12] Mollica RF, Sarajlic N (2001). Longitudinal study of psychiatric symptoms,disability, mortality and emigration among Bosnian refugees. JAMA..

[13] Silove D, Steel Z (2007). The impact of the refugee decision on the trajectory of PTSD, anxiety, and depressive symptoms among asylum seekers: a longitudinal study. Am J Disaster Med.

[14] Steel Z, Silove D (2002). Long-term effect of psychological trauma on the mental health of Vietnamese refugees resettled in Australia: a population-based study. Lancet..

[15] Harris M, Zwar N (2005). Refugee health. Aust Fam Physician.

[16] Silove D, Steel Z (2002). The impact of torture on post-traumatic stress symptoms in war affected Tamil refugees and immigrants. Compr Psychiatry.

[17] Kisely S, Stevens M (2002). Health issues of asylum seekers and refugees. ANZJPH.

[18] Schweitzer R, Melville F (2006). Trauma, post-migration living difficulties, and social support as predictors of psychological adjustment in resettled Sudanese refugees. Aust NZ J Psychiat.

[19] Lindencrona F, Ekblad S (2008). Mental health of recently resettled refugees from the Middle East in Sweden: the impact of pre-resettlement trauma, resettlement stress and capacity to handle stress. Soc Psych Psych Epid.

[20] Bogic M, Njoku A (2015). Long-term mental health of war-refugees: a systematic literature review. BMC Int Health Hum Right.

[21] Kirmayer LJ, Narasiah L (2011). Common mental health problems in immigrants and refugees: general approach in primary care: Canadian Guidelines for Immigrant Health. CMAJ.

[22] Fozdar F, Hartley L (2013). Refugee resettlement in Australia: what we know and need to know. Refug Surv Q.

[23] Fenta H, Hyman I (2004). Determinants of depression among Ethiopian immigrants and refugees in Toronto. J Nerv Ment D.

[24] Schweitzer RD, Brough M (2011). Mental health of newly arrived Burmese refugees in Australia: contributions of pre-migration and post-migration experience. Aust NZ J Psychiat..

[25] Chen W, Ling L (2017). A. Building a new life in Australia: an analysis of the first wave of the longitudinal study of humanitarian migrants in Australia to assess the association between social integration and self-rated health. BMJ Open.

[26] Um M, Chi I (2015). Correlates of depressive symptoms among north Korean refugees adapting to south Korean society: the moderating role of perceived discrimination. Soc Sci Med.

[27] Ahearn NE (2002). Psychological wellness of refugees: issues in qualitative and quantitative research.

[28] Gifford SM, Bakopanos C (2007). Meaning or measurement? Researching the social contexts of health and settlement among newly-arrived refugee youth in Melbourne, Australia. J Ref Stud.

[29] Dowling A, Enticott J (2017). Measuring self-rated health status among resettled adult refugee populations to inform practice and policy - a scoping review. BMC Health Serv Res.

[30] Creswell JW (2003). Research design: qualitative, quantitative, and mixed methods approaches.

[31] Australian Institute of Family Studies. Building a new life in Australia: the longitudinal study of humanitarian migrants. Australian Government Department of Social Services. http://www3.aifs.gov.au/bnla/ Accessed 4 May 2018.

[32] Taskakkori A, Teddlie C (1998). Mixed methodology: combining qualitative and quantitative approaches.

[33] De Maio J, Silbert M (2014). Building a new life in Australia: introducing the longitudinal study of humanitarian migrants. Fam Matters.

[34] Cross-Denny B, Robinson M (2017). Using the social determinants of health as a framework to examine and address predictors of depression in later life. Ageing Int.

[35] Ware JE, Gandek B (1998). Overview of the SF-36 health survey and the international quality of life assessment (IQOLA) project. J Clin Epidemiol.

[36] Kessler RC, Andrews G (2002). Short screening scales to monitor population prevalences and trends in non-specific psychological distress. Psychol Med.

[37] Bessaha M (2015). Factor structure of the Kessler psychological distress scale (K6) among emerging adults. Res Social Work Prac.

[38] Hansen M, Andersen TE (2010). PTSD-8: a short PTSD inventory. Clin Pract Epidemiol Ment Health.

[39] Silove D, Vijaya M (2007). Screening for depression and PTSD in a Cambodian population unaffected by war: Comparing the Hopkins Symptom Checklist and Harvard Trauma Questionnaire with the Structured Clinical Interview. J Nerv Ment Dis.

[40] Raudenbush SW, Bryk AS (2002). Hierarchical linear models.

[41] Corp S. Stata statistical software: release 14. SE 14.2 ed. College Station, TX:StataCorp LP; 2015.

[42] Glaser B, Strauss A (1967). The discovery of grounded theory: strategies for qualitative research.

[43] Miller KE, Worthngton GJ (2002). Bosnian refugees and the stressors of exile: a narrative study. Am J Orthop.

[44] Creswell JW, Plano Clark V (2007). Designing and conducting mixed methods research.

[45] Renner W, Senft B (2013). Predictors of unemployment in refugees. Soc Behav Personal.

[46] Lott JP (2005). Module three: vulnerable/special participant populations. Dev World Bioeth.

